# Pharmacovigilance and clinical insights into perindopril-induced thrombocytopenia: a case report of diagnostic challenges and therapeutic alternatives

**DOI:** 10.1186/s43044-026-00737-4

**Published:** 2026-04-28

**Authors:** Mochamad Faishal Riza, Samuel Sudanawidjaja, Ronald Torang Marsahala Panggabean, Isnaini Isnaini, Lely Puspita Candra Dewi, Andrianus Oktovianto, Deo Idarto, Vincent Aurelius Gonaldy, Alamanda Sofania, Marselina Marselina

**Affiliations:** 1 Cardiovascular Department, Moh Soewandhie Teaching Hospital, Ciputra University, Surabaya, Indonesia; 2 Nadhatul Ulama University, Surabaya, Indonesia; 3 School of Medicine, Ciputra University, Surabaya, Indonesia

**Keywords:** Perindopril, Thrombocytopenia, Angiotensin-converting enzyme inhibitors, Drug-induced thrombocytopenia, Case report

## Abstract

**Introduction and objectives:**

Perindopril-induced thrombocytopenia is a rare but potentially serious adverse drug reaction. It remains underrecognized in clinical practice, particularly in elderly patients with cardiovascular disease. This case highlights the importance of timely diagnosis and intervention in drug-induced hematologic complications.

**Case report:**

A 73-year-old woman with a history of percutaneous coronary intervention presented with unstable angina. During hospitalization, she exhibited an acute and unexplained decline in platelet count a week after the initiation of perindopril. Infectious causes, autoimmune disorders, and bone marrow dysfunction were systematically excluded. A medication review revealed recent perindopril initiation, and its discontinuation led to progressive platelet recovery. The strong temporal relationship confirmed perindopril-induced thrombocytopenia as the likely etiology (Table 1).

**Discussion:**

The immune-mediated destruction of platelets is proposed as the underlying mechanism, similar to other drug-induced thrombocytopenias. This case underscores the importance of systematic diagnostic evaluation, routine hematologic monitoring, and pharmacovigilance in patients receiving angiotensin-converting enzyme inhibitors. Alternative antihypertensive strategies, such as angiotensin receptor blockers, may be preferable in susceptible individuals.

**Conclusion:**

Perindopril-induced thrombocytopenia should be considered in patients presenting with unexplained platelet decline. Early recognition, drug discontinuation, and close monitoring are crucial in preventing complications. Further research is needed to refine diagnostic criteria and develop safer prescribing guidelines.

## Introduction

A 73-year-old woman with a history of percutaneous transluminal coronary angioplasty (PTCA) in June 2023 presented with unstable angina pectoris. During hospitalization, she exhibited an acute, unexplained decline in platelet count, prompting a thorough diagnostic workup. Infectious etiologies, including dengue fever, were ruled out, and peripheral blood smear analysis demonstrated no morphological abnormalities. Given the absence of other potential causes, a detailed medication review was undertaken, revealing a recent transition from captopril to perindopril. The temporal association between perindopril initiation and thrombocytopenia strongly implicated the drug as the causative factor. Upon discontinuation, platelet counts progressively normalized, further reinforcing this causal link.

This case underscores the necessity of heightened clinical vigilance for drug-induced hematologic disorders, particularly in elderly patients with multiple comorbidities. While angiotensin-converting enzyme (ACE) inhibitors are widely prescribed for cardiovascular disease, their potential to induce thrombocytopenia remains underrecognized. Early identification and prompt withdrawal of the offending agent are essential to mitigate hematologic complications and optimize patient outcomes.

## Background

Perindopril-induced thrombocytopenia is an uncommon but clinically significant adverse drug reaction that warrants careful consideration, particularly in elderly patients with complex cardiovascular histories. Thrombocytopenia, defined as a reduction in platelet count below the normal reference range, can predispose patients to bleeding complications and may signal an underlying hematologic disorder or drug-related toxicity.

Perindopril, an angiotensin-converting enzyme (ACE) inhibitor, is widely prescribed for hypertension and coronary artery disease (CAD). While effective, its potential hematologic adverse effects, particularly thrombocytopenia, remain underrecognized [9, 16]. Thrombocytopenia, characterized by a significant platelet decline, increases bleeding risk and complicates CAD management (Shirley & McCormack, 2015). The incidence of ACE inhibitor-induced thrombocytopenia is estimated at 0.2% to 4.6%, necessitating vigilant monitoring in high-risk populations [10, 12]. Understanding immune-mediated and toxic mechanisms is essential for optimizing patient safety [16].

Although relatively uncommon, perindopril-induced thrombocytopenia presents a clinically significant challenge in cardiovascular treatment. It complicates CAD therapy by increasing hemorrhagic risks, requiring adjustments in management strategies [9, 16]. Given its underreported nature, further research is needed to elucidate the risks and mechanisms [10, 12].

A literature review highlights key studies linking ACE inhibitors to hematologic complications. Periša et al., [16] associate perindopril with thrombotic microangiopathy (TMA), indicating microvascular damage. Garbe et al. [10] identify perindopril as a potential cause of drug-induced immune thrombocytopenia (DITP), emphasizing comprehensive patient evaluations. Elliott and Bistrika [8], stress long-term hematologic monitoring for ACE inhibitor recipients. Pharmacodynamic studies suggest that while ACE inhibitors improve cardiovascular outcomes, they may impair hematologic stability, particularly in elderly patients with polypharmacy [4, 20].

This case was selected due to its clinical importance and its contribution to understanding perindopril-induced thrombocytopenia. Given its rarity and potential severity, early recognition and intervention are crucial [9, 16]. This report underscores the necessity of hematologic monitoring in elderly patients and supports pharmacovigilance efforts, advocating alternative therapies such as angiotensin receptor blockers (ARBs) for high-risk individuals [10, 12].

In this case, a 73-year-old woman was admitted for unstable angina pectoris following a previous PTCA. Her clinical course was complicated by an unexpected and precipitous decline in platelet levels, necessitating an extensive differential diagnosis to exclude primary hematologic, infectious, and autoimmune causes. Despite comprehensive investigations, no alternative etiology was identified, leading to the hypothesis of a drug-induced mechanism. A meticulous review of her pharmacological history revealed a recent switch from captopril to perindopril. Given the temporal relationship between drug initiation and platelet decline, coupled with the subsequent normalization of platelet counts upon discontinuation, a diagnosis of perindopril-induced thrombocytopenia was established. Although ACE inhibitors are widely used for hypertension and cardiovascular disease management, their hematologic adverse effects, including thrombocytopenia, remain underreported and poorly understood.

This case highlights the importance of routine hematologic monitoring when initiating ACE inhibitors, particularly in older adults with polypharmacy. Recognizing and promptly addressing drug-induced thrombocytopenia is crucial to prevent severe complications and ensure optimal patient safety.

## Case presentation

### Initial presentation

A 73-year-old woman presented to the emergency department with acute chest pain that began earlier that morning. The pain occurred during mild activity and was associated with shortness of breath. It was described as a crushing, pressure-like sensation across the entire chest, with an 8 out of 10 intensities on the pain scale. The intermittent episodes lasted approximately 20 min and had an unpredictable onset. The symptoms were not alleviated by rest, and no associated manifestations such as nausea, vomiting, diarrhea, cough, or fever were associated. Her past medical history was notable for atherosclerotic heart disease with chronic ischemic heart disease, necessitating percutaneous coronary intervention (PCI) in June 2023. Additionally, she had a four-year history of hypertension with hypertensive heart disease but without congestive heart failure, as well as dyslipidemia and primary gonarthrosis. Perindopril therapy was initiated in 27th February 2025 as part of her post-PCI management. The patient reported no history of alcohol consumption, maintained no specific dietary restrictions, and had no known history of autoimmune diseases, chronic aspirin or ibuprofen use, or vitamin deficiencies. Her family history was unremarkable for cardiovascular or hematologic disorders.

### Physical examination

**Fig. 1:**
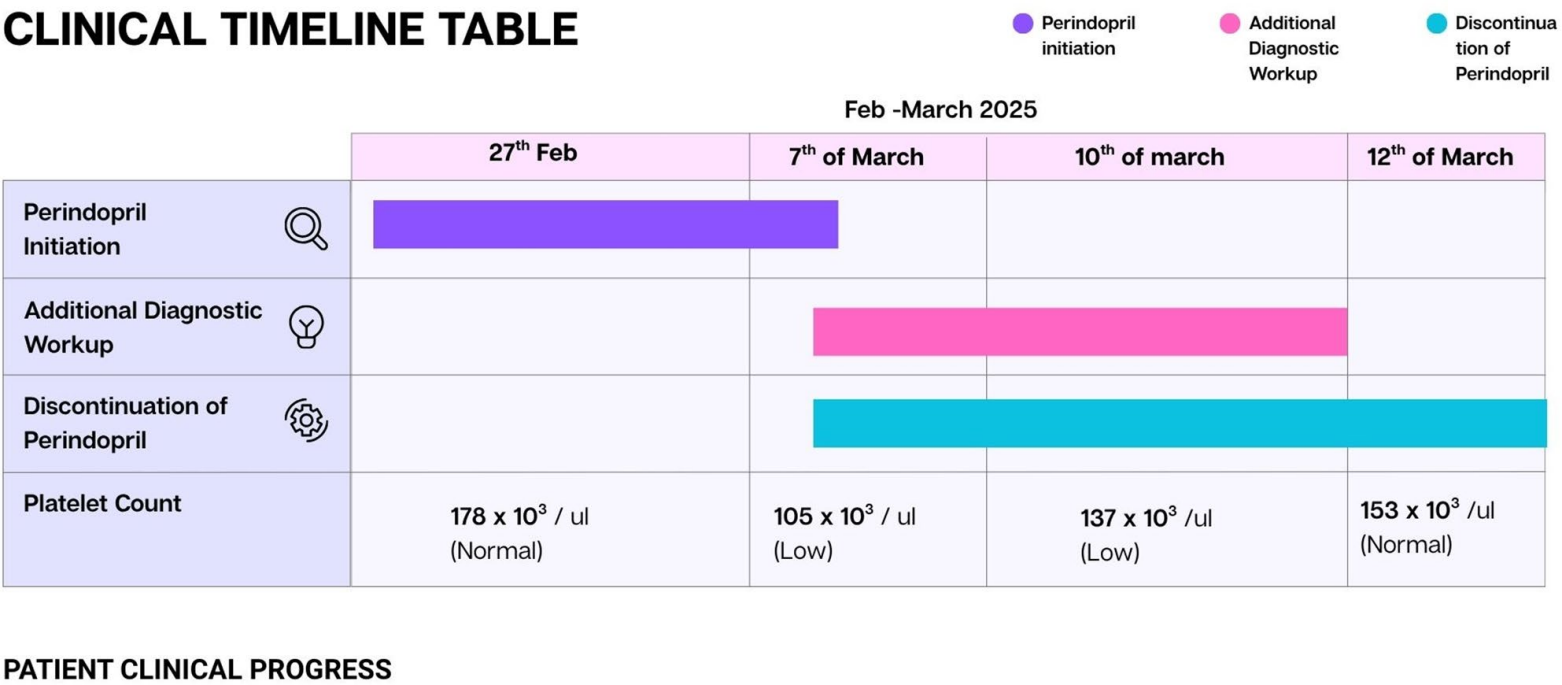
Clinical Timeline During Patient Hospitalization

Upon arrival, the patient was fully conscious and alert, with a Glasgow Coma Scale score of 15 (E4V5M6). Her vital signs were as follows: blood pressure of 146/83 mmHg, heart rate of 90 beats per minute, respiratory rate of 22 breaths per minute, oxygen saturation of 96% on room air, and body temperature of 36 °C. A comprehensive systemic examination revealed no signs of anemia, jaundice, or cyanosis. The respiratory assessment indicated symmetrical chest expansion with normal vesicular breath sounds and no adventitious sounds. The cardiovascular examination demonstrated a regular S1 and S2, with no murmurs or gallops. The abdominal assessment was unremarkable, with a soft, non-tender abdomen, normal bowel sounds, and no evidence of hepatosplenomegaly. Peripheral examination showed good perfusion, warm and dry extremities, capillary refill time of less than two seconds, and no signs of peripheral edema (Fig. 1).

### Investigations

Clear clinical table during our process of initial administration and subsequent discontinuation of perindopril, alongside with exclusion of alternative etiologies provided below, alongside the result of additional diagnostic workup in Table 1.

**Table 1:** .

Date	Platelet level	Decision
27th February 2025	178 × 10^3^/ µL	- Initiation of Perindopril
7th March 2025	105 × 10^3^/ µL	- Discontinuation of Perindopril - Excluding any possibilities of other thrombocytopenic causes (Complete blood count): - Neutrophilia (71%), Lymphocytopenia (18%), Monocytosis (8.2%), while Haemoglobin, Erythrocytes indices, and mean platelet volumes within normal limits. - Microscopic examination of peripheral blood smear shows normochromic normocytic red blood cells, mild leucocytosis, and no blast cells. There were no giant platelets or morphological abnormalities suggestive of a bone marrow disorder - IgM of Dengue Fever shows negative (excluding one of the most common tropical disease to cause rapid thrombocytopenia) - Coagulation profile : Prothrombin time (PT), Activated partial thromboplastin time (APTT), and international normalized ratio (INR) were within normal limits. - Cardiac Biomarkers: Creatinine kinase-MB (CKMB) was within the normal range at 3 ng/ml - Renal and Liver Function tests: All within normal limits.
10th March 2025	137 × 10^3^/ µL	- Further Monitoring of Platelet on a 3-day Basis (Following the Discontinuation of perindopril), Reticulocyte elevated at 3.03%
12th March 2025	153 × 10^3^/ µL	- Prior to discharge of patient, platelet was back to normal count.

The temporal association between perindopril initiation and the decline in platelet count, followed by platelet recovery upon drug discontinuation, strongly implicated perindopril-induced thrombocytopenia. The absence of alternative etiologies further supported this diagnosis, emphasizing the importance of pharmacovigilance in elderly patients receiving ACE inhibitors.

### Differential diagnosis

Thrombocytopenia is a multifactorial condition requiring systematic evaluation. Given the acute platelet decline observed in this patient, various etiologies were considered, including drug-induced, infectious, autoimmune, hematologic, and physiological causes. After a thorough assessment, perindopril-induced thrombocytopenia was determined to be the most plausible diagnosis.

1. Drug-Induced Thrombocytopenia (DITP)

DITP is a recognized adverse reaction to medications, including ACE inhibitors, antiplatelet agents, and anticoagulants [10]. The reported incidence ranges from 0.2% to 4.6%. In this case, the absence of other likely drug culprits and the rapid platelet recovery following perindopril cessation strongly supported DITP as the underlying cause.

2. Infectious Causes

Dengue fever, an endemic vector-borne disease, was considered due to its strong association with thrombocytopenia. However, negative serologies (IgM, IgG), the absence of dengue-related clinical signs, and platelet normalization post-drug withdrawal excluded this possibility [7].

3. Thrombotic Microangiopathies (TTP)

TTP presents with thrombocytopenia, hemolysis, and organ dysfunction. Drug-induced TTP is associated with antihypertensives, including ACE inhibitors [8]. However, normal LDH levels, absence of schistocytes, and no evidence of neurologic or renal dysfunction ruled out TTP.

4. Autoimmune Thrombocytopenia

Autoimmune disorders such as ITP and SLE can cause platelet destruction [17]. However, negative autoimmune markers and the resolution of thrombocytopenia upon perindopril discontinuation made an autoimmune cause unlikely.

5. Bone Marrow Disorders

Aplastic anemia, leukemia, and myelodysplastic syndromes can cause thrombocytopenia. In this case, normal hemoglobin levels (13.7 g/dL), a preserved reticulocyte count (3.03%), and an unremarkable peripheral smear effectively ruled out marrow failure [12].

6. Hypersplenism

Splenic sequestration due to liver disease can result in thrombocytopenia [9]. However, normal liver function tests and the absence of splenomegaly excluded hypersplenism.

7. Pseudothrombocytopenia

This laboratory artifact results from platelet clumping in EDTA-treated samples (藤井, 2011). Repeated platelet counts confirmed true thrombocytopenia, eliminating pseudothrombocytopenia.

### Treatment and management

The primary strategy in managing perindopril-induced thrombocytopenia is promptly discontinuing the offending agent, followed by close hematologic monitoring and supportive care. In this case, the strong temporal association between perindopril initiation and thrombocytopenia necessitated immediate withdrawal to prevent further platelet decline and mitigate hemorrhagic complications.

Following perindopril cessation, the patient demonstrated progressive platelet recovery, reinforcing the drug’s role in inducing thrombocytopenia. This aligns with previously documented cases, where platelet normalization typically occurs within days to weeks after discontinuation of ACE inhibitors. Although the precise immunopathogenic mechanism remains unclear, it is hypothesized that drug-dependent antibodies targeting platelet glycoproteins may contribute to immune-mediated destruction, necessitating careful consideration when reintroducing ACE inhibitors in affected individuals.

The patient’s therapeutic approach included regular platelet monitoring and risk assessment for bleeding complications. Given the absence of clinically significant hemorrhage and preserved hemostatic function, platelet transfusion was not required. However, in cases of severe thrombocytopenia (< 20,000/µL) or active bleeding, platelet transfusions and immunosuppressive therapy (such as corticosteroids or intravenous immunoglobulin) may be warranted.

Candesartan, an angiotensin receptor blocker (ARB), was introduced as a safer alternative to ACE inhibitors to maintain optimal cardiovascular risk management. ARBs have been associated with a lower risk of immune-mediated thrombocytopenia while providing comparable renin-angiotensin-aldosterone system (RAAS) inhibition. This substitution is supported by existing literature advocating for ARBs in patients with a history of ACE inhibitor-induced hematologic reactions [2, 6].

The patient’s concurrent medications, including bisoprolol, amlodipine, atorvastatin, and antianginal therapy, were continued to ensure comprehensive secondary prevention in the context of coronary artery disease (CAD). Additionally, nonsteroidal anti-inflammatory drugs (NSAIDs) and other platelet-affecting agents were avoided to prevent exacerbation of thrombocytopenia.

This case underscores the importance of pharmacovigilance in patients receiving ACE inhibitors, particularly those with complex cardiovascular comorbidities. Routine laboratory surveillance and heightened clinical suspicion for drug-induced hematologic abnormalities are essential for early detection and prevention of adverse outcomes. Further research into the immunopathologic mechanisms underlying ACE inhibitor-induced thrombocytopenia is warranted to identify predictive risk factors and optimize prescribing practices in susceptible populations.

### Outcome and follow-up

Given the absence of other potential causes for thrombocytopenia, the temporal association between perindopril initiation and the drop in platelet count, and the recovery following drug discontinuation, a diagnosis of perindopril-induced thrombocytopenia was considered. The decision was made to discontinue perindopril on 07 March 2025, and the patient was closely monitored with serial platelet counts. By 12 March 2025, platelet levels had normalized without additional intervention. The patient remained hemodynamically stable and asymptomatic throughout the monitoring period. She was started on an alternative antihypertensive regimen and discharged with outpatient follow-up to ensure stable platelet counts and optimal cardiovascular management.

This case underscores the importance of considering drug-induced thrombocytopenia in patients receiving angiotensin-converting enzyme inhibitors, particularly when alternative causes have been excluded. Clinicians should maintain a high index of suspicion for this rare but potentially serious adverse effect to prevent unnecessary interventions and ensure timely management.

## Discussion

### Clinical implications of perindopril-induced thrombocytopenia

This case highlights the rare but significant risk of thrombocytopenia associated with perindopril use in a cardiovascular patient. The temporal association between perindopril initiation and platelet decline, followed by normalization after drug discontinuation, strongly implicates the drug as the causative agent. The absence of alternative explanations, including infections, autoimmune conditions, and bone marrow disorders, supports the diagnosis. Given that ACE inhibitor-induced thrombocytopenia is underreported, this case underscores the need for clinical vigilance, particularly in elderly patients with multiple comorbidities.

Patients susceptible to perindopril-induced thrombocytopenia typically exhibit specific demographic and clinical characteristics. Elderly individuals face an increased risk due to age-related physiological changes, polypharmacy, and declining renal function, which impact drug metabolism and predispose them to adverse reactions [16, 17]. Comorbidities such as hematological disorders, chronic renal disease, or the necessity for anticoagulant therapy further heighten vulnerability due to altered pharmacokinetics [3, 20]. Women may also have a higher predisposition to immune-mediated drug reactions, although data on gender differences in perindopril-induced thrombocytopenia remain limited [7, 14]. Additionally, genetic factors influence individual drug metabolism, with pharmacogenomic screening potentially identifying high-risk populations [3, 18]. Drug interactions, particularly with anticoagulants or antiplatelet agents, can exacerbate hematological complications, emphasizing the necessity of comprehensive medication reviews [21, 22]. Patients with chronic conditions such as diabetes, cardiovascular diseases, and autoimmune disorders may have altered platelet function, increasing their susceptibility to drug-induced hematologic abnormalities [12, 19]. Understanding these risk factors is crucial for patient monitoring and preventing severe hematological complications.

The precise mechanism by which ACE inhibitors induce thrombocytopenia remains unclear, though a primary hypothesis has been proposed, which mechanism is Immune-mediated-mechanism, where drug dependent antibodies may target platelet glycoprotein, leading to its destruction [2]. Foundational insights into immune-mediated Ace inhibitor-induced thrombocytopenia were provided by Ackroyd, 1998 aligning our case findings and suggesting a drug-dependent immune response causing thrombocytopenia, reinforcing Aster and Bougie [2].

Garbe et al. [10] estimate the incidence of ACE inhibitor-induced thrombocytopenia between 0.2% and 4.6%, highlighting its rarity but clinical significance. Pharmacovigilance data emphasize the unpredictable nature of hematologic toxicity in ACE inhibitors, necessitating proactive monitoring in at-risk populations.

This case reinforces the importance of recognizing drug-induced thrombocytopenia early and discontinuing the offending agent promptly to prevent complications. Drug discontinuation is a critical step, as evidenced by studies demonstrating platelet count recovery upon withdrawal of the suspected drug [11]. Laboratory confirmation of thrombocytopenia is essential, including a complete blood count (CBC) and a peripheral blood smear to exclude pseudothrombocytopenia. Further investigations, such as autoimmune tests like the direct antiglobulin test (DAT) and ADAMTS13 activity, can help differentiate immune-mediated thrombocytopenia from other causes [13].

Latest reported case is from 2021, when 48 years old women suffers from thrombotic microangiopathy (TMA) after undergoing combination therapy of perindopril/amlodipine with symptoms showing rapidly (2 days after initiation) with a platelet count nadir of 11 × 10^3^/µL. After discontinuation of perindopril alongside plasma exchange therapy and corticosteroids results in patient clinical improvement proven by normalization of platelet count, supporting causal relationship between perindopril and thrombocytopenia [5].

Given the patient’s history of coronary artery disease (CAD) and percutaneous coronary intervention (PCI), routine laboratory surveillance was crucial in detecting thrombocytopenia before the onset of clinical symptoms.

## Conclusion and future directions

This case reinforces evidence linking ACE inhibitors, particularly perindopril, to drug-induced thrombocytopenia, necessitating a multifaceted approach for early detection and management. Routine platelet monitoring in high-risk patients, such as the elderly and those with renal impairment, can facilitate early intervention [4]. Screening tools, including genetic testing, may help identify susceptible individuals before therapy initiation [6, 10]. Immediate drug discontinuation upon suspicion of thrombocytopenia is critical to prevent complications [4]. In severe cases, supportive care may be required, including platelet transfusions and corticosteroids [15]. Strengthening pharmacovigilance through adverse drug reaction tracking and electronic health records enhances early detection [1]. Educating healthcare providers and patients on recognizing hematologic side effects ensures better clinical outcomes [4, 12]. Future research should explore safer antihypertensive alternatives and the long-term hematologic impact of ACE inhibitor-induced thrombocytopenia [17].

Strength of this case report it is that it highlights an underrecognized, potentially serious adverse reaction- perindopril-induced thrombocytopenia in elderly patient, clinical relevance and novelty increases as few documented pharmacovigilance literatures was documenter prior to similar cases. Systematic exclusion of alternative causes (infectious, autoimmune, marrow disorder, including pseudothrombocytopenia) was done and ensures deetailed laboratory data presented to support the diagnosis, weakness of this case it is the single case limitation, findings from one patient cannot establish incidence or risk factors, and the inability to specify other populations, this case is specific for elderly patients with comorbid it may differ in younger or less complex patients.

## Learning points/take home messages

1. Recognizing Perindopril-Induced Thrombocytopenia. Though rare, perindopril-induced thrombocytopenia can pose serious risks, especially in elderly patients. A clear temporal association between drug initiation, platelet decline, and recovery upon discontinuation is key to diagnosis.
2. Refining Diagnostic Approaches. Systematic platelet monitoring, peripheral blood smear analysis, and autoimmune testing help rule out infections, bone marrow disorders, and other causes. Pharmacogenomic screening may further aid in identifying high-risk individuals.
3. Optimizing Management Strategies. Upon suspicion of thrombocytopenia, immediate discontinuation of perindopril is crucial. ARBs provide a safer antihypertensive alternative in affected patients.
4. Enhancing Pharmacovigilance. Strengthening pharmacovigilance, integrating electronic health records, and global adverse drug reaction reporting can improve early detection and patient safety.
5. Advancing Research and Safe Prescribing. Further studies are needed to elucidate mechanisms, assess genetic predispositions, and refine antihypertensive treatment guidelines for at-risk populations.

## Perspective

Patient’s perspective: I had my heart procedure in June 2023 and was given perindopril in December, but I only started taking it regularly in February 2025. I didn’t feel any different—no pain, no dizziness, nothing unusual. Then in March, my doctor told me my platelets had dropped a lot. I was surprised because I felt completely fine. They stopped the perindopril, and my blood tests improved after some time. I never thought a blood pressure pill could cause this. If the doctor hadn’t checked my blood, I wouldn’t have known anything was wrong.

Family’s Perspective: She was taking her medicines as usual, and we never noticed any problems—no bleeding, no bruising. When the doctor said her platelets were very low, it didn’t make sense because she looked and felt fine. Luckily, they caught it early. This made us realize how important routine check-ups are, even without symptoms. If they hadn’t checked, we wouldn’t have known anything was happening inside her body.

## Data Availability

The datasets analysed during the current study are not publicly available due to risks of breaching personal privacy of the patient but are available from the corresponding author on reasonable request.

## Abbreviations

ACE: Angiotensin Converting Enzyme
ARB: Angiotensin receptor blocker
CAD: Coronary artery disease
CBC: Complete blood count
CK-MB: Creatinine Kinase- Myocardial Band
DAT: Direct antiglobulin test
DITP: Drug-induced immune thrombocytopenia
EDTA: Ethylenediaminetetraacetic Acid
GCS: Glasgow Coma Scale
ITP: Immune Thrombocytopenic Purpura
INR: International normalized ratio
LDH: Lactate Dehydrogenase
MPV: Mean platelet volume
NSAIDs: Nonsteroidal anti-inflammatory drugs
PCI: Percutaneous coronary intervention
PT: Prothrombin time
PTCA: Percutaneous transluminal coronary angioplasty
RAAS: Renin-angiotensin-aldosterone system
SLE: Systemic Lupus Erythematosus
TMA: Thrombotic microangiopathy
TTP: Thrombotic thrombocytopenic purpura
